# Association of the shuffling of *Streptococcus pyogenes *clones and the fluctuation of scarlet fever cases between 2000 and 2006 in central Taiwan

**DOI:** 10.1186/1471-2180-9-115

**Published:** 2009-06-01

**Authors:** Chien-Shun Chiou, You-Wun Wang, Pei-Ling Chen, Wan-Ling Wang, Ping-Fuai Wu, Hsiao-Lun Wei

**Affiliations:** 1 The Central Region Laboratory, Center for Research and Diagnostics, Centers for Disease Control, Taichung City 40855, Taiwan; 2 Institute of Medicine, Chung Shan Medical University, Taichung City 40201, Taiwan; 3 The Second Division, Centers for Disease Control, Taichung City 40855, Taiwan

## Abstract

**Background:**

The number of scarlet fever occurrences reported between 2000 and 2006 fluctuated considerably in central Taiwan and throughout the nation. Isolates of *Streptococcus pyogenes *were collected from scarlet fever patients in central Taiwan and were characterized by *emm *sequencing and a standardized pulsed-field gel electrophoresis (PFGE) method. National weekly report data were collected for investigating epidemiological trends.

**Results:**

A total of 23 *emm *types were identified in 1,218 *S. pyogenes *isolates. The five most prevalent *emm *types were *emm*12 (50.4%), *emm*4 (23.2%), *emm*1 (16.4%), *emm*6 (3.8%) and *emm*22 (3.0%). PFGE analysis with *Sma*I suggested that, with a few exceptions, strains with a common *emm *type belonged to the same clone. There were two large *emm*12 clones, one with DNA resistant to cleavage by *Sma*I. Each prevalent *emm *clone had major PFGE strain(s) and many minor strains. Most of the minor strains emerged in the population and disappeared soon after. Even some major strains remained prevalent for only 2–3 years before declining. The large fluctuation of scarlet fever cases between 2000 and 2006 was associated with the shuffling of six prevalent *emm *clones. In 2003, the dramatic drop in scarlet fever cases in central Taiwan and throughout the whole country was associated with the occurrence of a severe acute respiratory syndrome (SARS) outbreak that occurred between late-February and mid-June in Taiwan.

**Conclusion:**

The occurrences of scarlet fever in central Taiwan in 2000–2006 were primarily caused by five *emm *types, which accounted for 96.8% of the isolates collected. Most of the *S. pyogenes *strains (as defined by PFGE genotypes) emerged and lasted for only a few years. The fluctuation in the number of scarlet fever cases during the seven years can be primarily attributed to the shuffling of six prevalent *emm *clones and to the SARS outbreak in 2003.

## Background

*Streptococcus pyogenes *(Group A streptococcus) is a common pathogen responsible for a number of human suppurative infections, including pharyngitis, impetigo, pyoderma, erysipelas, cellulitis, necrotizing fasciitis, toxic streptococcal syndrome, scarlet fever, septicemia, pneumonia and meningitis. It also causes non-suppurative sequelae, including acute rheumatic fever, acute glomerulonephritis and acute arthritis [1]. Scarlet fever, characterized by a sore throat, skin rash and strawberry tongue, is most prevalent in school children aged four to seven years old. This disease was listed as a notifiable disease in Taiwan until 2007; as such, all cases of scarlet fever had to be reported to the public heath department. According to our records, however, only 9% of the medical centers, regional hospitals and district hospitals in central Taiwan reported cases of scarlet fever to the health authorities between 1996 and 1999. The number of scarlet fever cases is therefore likely to be significantly underreported. Scarlet fever outbreaks frequently occur in young children at day-care centers, kindergartens and elementary schools [2,3] and also occur in adults upon exposure to contaminated food [4].

Genotyping bacterial isolates with various methods is frequently used to compare the genetic relatedness of bacterial strains and provides useful information for epidemiological studies. In a previous study, we used *emm *(gene of M protein) sequencing [5], vir typing [6] and pulsed-field gel electrophoresis (PFGE) typing to analyze a collection of streptococcal isolates from scarlet fever patients and used these data to build a DNA fingerprint and *emm *sequence database for long-term disease surveillance [7]. Vir typing has since been abandoned in our lab because it has lower discriminatory power than PFGE and the protocol is difficult to standardize with conventional agarose gel electrophoresis. In contrast, the PFGE protocol for *S. pyogenes *has been standardized in our laboratory, and a second enzyme, *SgrA*I, has been found to replace *Sma*I for analysis of strains with DNA resistant to *Sma*I digestion [7]. Since PFGE is highly discriminative and *emm *sequencing provides unambiguous sequence information regarding *emm *type, we adopted these two genotyping methods to characterize streptococcal isolates and build a *Streptococcus pyogenes *DNA fingerprint and sequence database for the long-term study of scarlet fever and other streptococcal diseases.

The number of scarlet fever cases in central Taiwan fluctuated greatly between 2000 and 2006. Relative to the number of scarlet fever occurrences in 2000, occurrences increased in 2001 and doubled in 2002, but dramatically dropped in 2003. The number of occurrences increased again since 2004. In this study, we characterized 1,218 isolates collected between 2000–2006 by *emm *sequencing and PFGE. The bacterial genotyping data and the epidemiological data collected via the Notifiable Disease Reporting System (established by Taiwan Centers for Disease Control (Taiwan CDC)) were used to examine the significant fluctuation in the number of scarlet fever cases between 2000 and 2006.

## Results

### Epidemiological trend of scarlet fever

**Figure 1:**
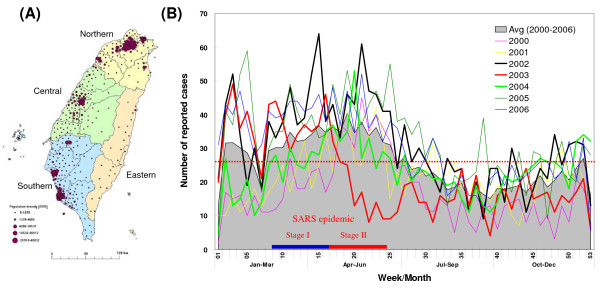
**(A) Map of Taiwan and population density (B) National weekly reported cases of scarlet fever between 2000 and 2006**. The total average throughout 2000–2006 is indicated by a red dashed line. The two stages of the SARS epidemic in 2003 are marked by blue (stage I) and red (stage II) bars.

Taiwan is an island country populated by 22.9 million people, most of whom reside in the western region (Figure 1A). The population in northern, central, southern, and eastern areas is 10.2, 5.7, 6.4 and 0.6 million, respectively. Nationwide information for all notifiable diseases has been systematically collected since 2000. For accurate analysis, the number of confirmed scarlet fever cases was adjusted by multiplying the number of reported cases and the specimen positive rate. The total, adjusted number of confirmed cases throughout the whole country increased from 716 cases in 2000 to 1,258 in 2002, but dramatically dropped to 771 in 2003 (Table 1). This number increased again in 2004 and, in 2005, reached the high levels seen in 2002. However, the number of cases slightly declined again in 2006. In central Taiwan, the epidemiological trend was similar to the national profile, but fluctuated more dramatically between 2000 and 2004. While the number of scarlet fever cases was 142 in 2000, this number doubled in 2002 but then dropped in 2003 to the levels seen in 2000 (Table 1). The number of cases increased again in 2004 and, in 2006, reached the levels seen in 2002. The number of cases in 2006 was greater than that in 2005 and differed from the national trend. The number of cases in central Taiwan accounted for 18% to 24% of cases throughout the whole country.

**Table 1:** Reported and adjusted confirmed scarlet fever cases in the whole country and in central Taiwan from 2000 to 2006.

Category	2000	2001	2002	2003	2004	2005	2006
Nationwide							
Reported cases (A)	924	1143	1655	1162	1254	1713	1635
Specimens collected (B)	659	792	1359	964	1100	1614	1594
Sampling rate % (B/A)	71%	69%	82%	83%	88%	94%	97%
Laboratory confirmed cases (C)	511	574	1033	640	759	1132	1130
Positive rate % (C/B)	78%	72%	76%	66%	69%	70%	71%
Adjusted confirmed cases (A × C/B)	716	828	1258	771	865	1201	1159
							
Central region							
Reported cases (A)	161	218	332	197	231	307	357
Specimens collected (B)	129	199	307	182	219	305	355
Sampling rate % (B/A)	80%	91%	92%	92%	95%	99%	99%
Laboratory confirmed cases (C)	114	146	260	135	156	216	272
Positive rate % (C/B)	88%	73%	85%	74%	71%	71%	77%
Adjusted confirmed cases (A × C/B)	142	160	281	146	165	217	274
% of central region/nationwide	20%	19%	22%	19%	19%	18%	24%
Isolates collected for analysis	139	154	273	122	115	174	241

The profiles of weekly reported cases revealed that scarlet fever was more prevalent in the winter and spring seasons (2^nd ^– 25^th ^weeks) in 2000–2006. However, there was a remarkable decrease in the number of cases in the 6^th ^and 7^th ^weeks (Figure 1B). This decrease may be due to the long holiday of the traditional lunar New Year and winter break from school, as it is usually from late-January to mid-February (4^th ^– 7^th ^weeks). The weekly reported number of scarlet fever cases in 2002 was mostly higher than the weekly average from 2000 to 2006 (Figure 1B). In 2003, except in the 11^th ^week, the number of weekly reported cases in the first 16 weeks was greater than the average. Furthermore, the number of cases between the 4^th ^and 9^th ^weeks was even higher than that in 2002. After the 16^th ^week, the number of cases in 2003 was below the overall average and was significantly decreased from the 17^th ^to 24^th ^week (mid-April to mid-June). A lower level of reported cases lasted until the first half of year 2004. In early 2003, a severe acute respiratory syndrome (SARS) outbreak occurred in Taiwan. There were two stages for the SARS epidemic: stage I occurred from late-February to mid-April (9^th ^– 16^th ^week), with scattered sporadic cases, and stage II occurred between mid-April and mid-June (17^th ^– 24^th ^week), with severe nosocomial infections in several hospitals. The dramatic decline of scarlet fever notifications in 2003 occurred during the stage II period of the SARS epidemic.

### Distribution of *emm *types among isolates collected in central Taiwan

For each year between 2000 and 2006, 115 to 273 isolates were collected for genotyping in central Taiwan (Table 1). A total of 1,218 isolates were characterized to investigate the distribution of *emm *types. In total, 23 *emm *types were identified in the isolates. The five most prevalent *emm *types, accounting for 96.8% of the collection, were *emm*12 (50.4%), *emm*4 (23.2%), *emm*1 (16.4%), *emm*6 (3.8%) and *emm*22 (3.0%) (Table 2). *emm*12 was the predominant type found between 2000–2001, accounting for 87.1% and 57.1% of the total isolates in 2000 and 2001, respectively. It became the predominant type again in 2005 and 2006, accounting for 69.3% of the isolates in 2006. *emm*1 was predominant in 2002, *emm*4 was most prevalent in 2003 and 2004, and *emm*6 emerged in 2001 but was not detected again after 2003.

**Table 2:** Distribution of *emm *types in *Streptococcus pyogenes *isolates collected in central Taiwan from 2000 to 2006

*emm *Type	Number (%) of isolates in year							Total
		
	2000	2001	2002	2003	2004	2005	2006	
*emm*12	121 (87.1)	88 (57.1)	64 (23.4)	17 (13.9)	45 (39.1)	112 (64.4)	167 (69.3)	614 (50.4)
*emm*4	11 (7.9)	21 (13.6)	58 (21.2)	54 (44.3)	57 (49.6)	39 (22.4)	43 (17.8)	283 (23.2)
*emm*1	4 (2.9)	35 (22.7)	111 (40.7)	26 (21.3)	9 (7.8)	10 (5.7)	5 (2.1)	200 (16.4)
*emm*6	0 (0.0)	6 (3.9)	26 (9.5)	14 (11.5)	0 (0.0)	0 (0.0)	0 (0.0)	46 (3.8)
*emm*22	1 (0.7)	1 (0.6)	2 (0.7)	1 (0.8)	3 (2.6)	10 (5.7)	18 (7.5)	36 (3.0)
Other*	2 (1.4)	3 (1.9)	12 (4.4)	10 (8.2)	4 (3.5)	0 (0.0)	8 (3.3)	39 (3.2)

Total	139	154	273	122	115	174	241	1218

*18 *emm *types: *emm*2 (5 isolates), *emm*11 (11), *emm*28 (1), *emm*49 (5), *emm*58 (1), *emm*76 (1), *emm*77 (1), *emm*81 (1), *emm*82 (1), *emm*89 (3), *emm*92 (1), *emm*101 (1), *emm*102 (1), *emm*103 (1), *st*2904 (2), *st*5282 (1), *st*G485 (1), *st*IL103 (1)

### PFGE and *emm *genotypes

**Figure 2:**
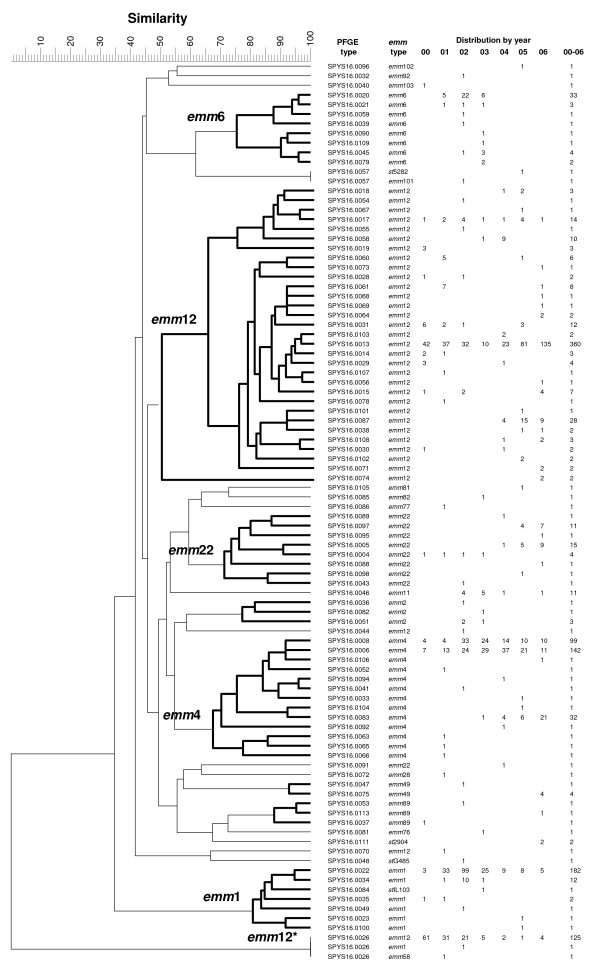
**Dendrogram constructed with PFGE-*Sma*I patterns, with their corresponding *emm *types and number of isolates obtained between 2000 and 2006**. The clustering analysis was performed with BioNumerics using the UPGMA algorithm and the value of Dice predicted similarity of two patterns at settings of 1% optimization and 0.7% position tolerance.

The 1,218 *S. pyogenes *isolates were analyzed by PFGE with *Sma*I to investigate the clonal relationship among the isolates. There were 127 isolates with DNA resistant to *Sma*I digestion, and their pattern (with only one DNA band) was referred to as a SPYS16.0026 PFGE-*Sma*I type. The 127 isolates with the SPYS16.0026 genotype were further analyzed by digestion with *SgrA*I. The genetic relatedness of the bacterial strains was evaluated by the levels of similarity among the PFGE-*Sma*I patterns. A dendrogram was constructed using the Unweighted Pair Group Method with Arithmatic mean (UPGMA) algorithm. The dendrogram revealed that all of the *emm*4 and *emm*6 isolates, as well as the majority of *emm*1 and *emm*22 isolates, were each distributed in a unique cluster. However, the *emm*12 isolates were located in two distinct clusters and two singletons (Figure 2). One of these clusters included 125 *emm*12 isolates that were resistant to *Sma*I digestion. Clustering analysis indicated that isolates with a common *emm *type were, in general, more closely related than those with different *emm *types. However, there were a few exceptions. Two strains with different *emm *types (*emm*101 and *st*5282) had indistinguishable PFGE-*Sma*I patterns, and a strain with a *st*IL103 type was located within the *emm*1 cluster (Figure 2). *st*IL103 is an allele of *emm*1 that lacks the codons encoding the mature M1 7–24 residues (http://www.cdc.gov/ncidod/biotech/strep/strepindex.htm; accessed on April 20^th^, 2009). Sequence analysis suggests that the *st*5282 strain could be derived from an *emm*101 strain via *emm *gene recombination, as the sequence for the first 26 codons of the *st*5282 gene were identical to that for the *emm*101 gene. Two *emm*12 and one *emm*22 isolates were distant from the major *emm*12 and *emm*22 clusters (Figure 2). The 127 *Sma*I-resistant isolates were identified to be of *emm*12, *emm*1 or *emm*58 type.

In total, 94 *emm*:PFGE-*Sma*I genotypes were identified in the 1,218 isolates. Eight major *emm*:PFGE genotypes, *emm*1:SPYS16.0022 (14.9%), *emm*4:SPYS16.0006 (11.7%), *emm*4:SPYS16.0008 (8.1%), *emm*4:SPYS16.0083 (2.6%), *emm*6:SPYS16.0020 (2.7%), *emm*12:SPYS16.0013 (29.6%), *emm*12:SPYS16.0026 (10.3%) and *emm*12:SPYS16.0087 (2.3%), made up 82.2% of the 1,218 isolates. Five of the major *emm*:PFGE genotypes were detected throughout the seven years studied. In contrast, most *emm*:PFGE genotypes lasted for only 1–2 years; they emerged in the population and quickly disappeared.

**Figure 3:**
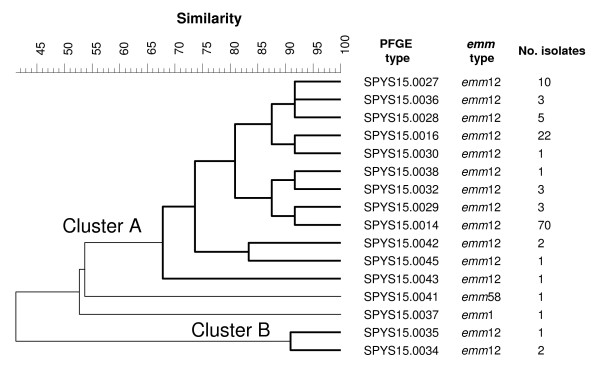
**Dendrogram constructed with PFGE-*SgrA*I patterns, with their corresponding *emm *types and number of isolates**. DNA from these isolates was resistant to *Sma*I digestion. The clustering analysis was performed with BioNumerics using the UPGMA algorithm and the value of Dice predicted similarity of two patterns at settings of 1% optimization and 0.7% position tolerance.

The 127 *Sma*I-resistant isolates were discriminated by PFGE with *SgrA*I into 14 *emm*12:PFGE-*SgrA*I, 1 *emm*1:PFGE and 1 *emm*58:PFGE types. The 125 *emm*12 isolates were distributed in two distinct clusters, A and B (Figure 3). Strains within cluster A were quite divergent, with the most divergent types sharing only 65% pattern similarity.

### Distribution of prevalent *emm *clones over time

**Figure 4:**
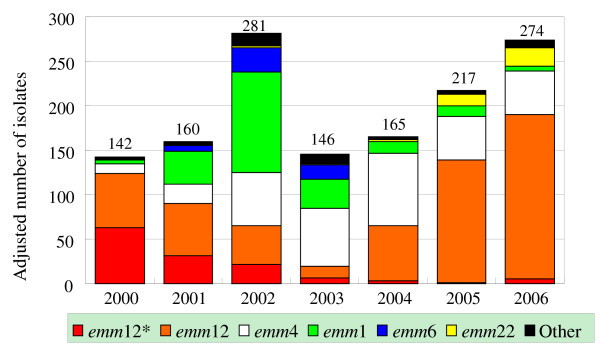
**Distribution of *emm *clones between 2000 and 2006**. The number of *Streptococcus pyogenes *isolates analyzed is adjusted according to the number of adjusted annual confirmed of cases.

In this study, a cluster of strains (as defined by PFGE types) having a common *emm *type and sharing higher PFGE pattern similarity than others with different *emm *types were considered to belong to a common *emm *clone. The *st*IL103 strain is an exception to this, as it shared high PFGE pattern similarity with the cluster of *emm*1 strains and was therefore considered to be part of the *emm*1 clone. Based on the groupings made by the PFGE patterns, six major *emm *(*emm*1, *emm*4, *emm*6, *emm*12, *emm*12* and *emm*22) clones were identified and are shown in Figure 2. The *emm*12* clone represents the *emm*12 strains with DNA resistant to *Sma*I digestion. The six major *emm *clones made up 96.5% of the 1,218 isolates. The adjusted number of the annual confirmed cases of scarlet fever in central Taiwan ranged from 142 to 282 between 2000 and 2006 (Table 1), and 115 to 273 isolates were collected each year for genotyping. The number of isolates genotyped was adjusted to the number of annual confirmed cases to investigate the association of the fluctuation of scarlet fever cases and the relative prevalence of the *emm *clones. As shown in Figure 4, the *emm*12* and *emm*12 clones were the most prevalent in 2000. The two clones declined over time and were at their lowest levels in 2003. The *emm*1 clone was the most prevalent in 2002 and the *emm*4 clone was predominant in 2003 and 2004. In 2001, although the number of *emm*12* and *emm*12 clones declined, the number of *emm*1 clones increased significantly. The total number of scarlet fever cases in 2002 was doubled that in 2000 and were primarily attributed to an increase in the number of the *emm*1, *emm*4 and *emm*6 clones. The number of cases in 2003 was considerably lower than that in 2002, likely due to a decline in all major clones except for *emm*4. The number of cases increased significantly again in 2005, and this increase is associated with a dramatic rise in the prevalence of the *emm*12 clone.

## Discussion

The cases of scarlet fever in central Taiwan from 2000 to 2006 were caused by *S. pyogenes *strains with a limited number of *emm *types (Table 2). In fact, five prevalent *emm *types represented 96.8% of the isolates causing scarlet fever during this time period. Of the 23 *emm *types isolated, 17 made up 99.4% of the isolates. These 17 types were among the 30 most common *emm *types that caused invasive streptococcal infections in the United States between 2000 and 2004. Twelve of these types accounted for 75.5% of the isolates characterized and were included in the proposed 26-valent vaccine (*emm *types 1, 1.2, 2, 3, 5, 6, 11, 12, 14, 18, 19, 22, 24, 28, 29, 33, 43, 59, 75, 76, 77, 89, 92, 94, 101, and 114) [8].

In our previous work on 179 *S. pyogenes *isolates collected in central Taiwan between 1996 and 1999, the five most common *emm *types in central Taiwan remained the same, but the frequency changed in the two time periods, 1996–1999 and 2000–2006 [7]. However, the prevalence and distribution of *emm *types could have geographic variation. Yan et al. [9] analyzed 77 *S. pyogenes *isolates collected from scarlet fever patients between 1993 and 2002 in southern Taiwan and found only three *emm *types among the isolates, with *emm*1 being the most prevalent type. Chen and colleagues characterized 830 isolates collected between 2001 and 2002 in northern Taiwan and found that the most frequent *emm *types were *emm*1 (29.2%), *emm*4 (24.1%), *emm*12 (19.0%), *emm*6 (15.8%), *st*IL103 (5.7%) and *emm*22 (1.9%) [10]. In our study, the most common *emm *types in 427 isolates collected in the same time period in central Taiwan were *emm*12 (35.6%), *emm*1 (34.2%), *emm*4 (18.5%), *emm*6 (7.5%) and *emm*11 (0.9%). *st*IL103 was present in northern Taiwan, but it was not found in the central region during the same time period. Thus, the distribution and frequency of *emm *types appear to be geographically varied even in such a small country. The geographic variation in the prevalence of *emm *clones may explain why the epidemiological trend of scarlet fever in 2006 in central Taiwan was different from that in the whole country.

The major *emm *types were further discriminated into a number of PFGE types, and clustering analysis of the PFGE patterns suggests that the *emm*1, *emm*6 and *emm*4 strains belong to a single clone. The *emm*12 strains belong to two major clones and two singletons, and *emm*22 strains belong to one major clone and one singleton (Figure 2). Thus, six *emm *clones caused most (96.5%) of the scarlet fever cases in central Taiwan during the seven year time period. The fluctuation of scarlet fever cases was associated with the shuffling of the prevalent *emm *clones (Figure 4). The finding that only a few prevalent M (*emm*) types caused most occurrences of scarlet fever in a specific location in a given year period, as well as the shuffling of predominant M types, has been observed in many epidemiological studies in the early 20^th ^century [11]. During major epidemics of streptococcal infections in previous years, only a few serotypes predominated, and the strains were rich in M protein, encapsulated and were highly virulent [11]. Type-specific immunity was important for preventing re-infection with the same M type. It is thought that the shuffling of predominant M types is due to the type-specific immunity, leading to the decline of infections with certain M types and the emergence of other virulent M types. In the present study, the prevalence of the *emm*12*, *emm*1 and *emm*6 clones both increased and decreased within one year. In contrast, the *emm*12 and *emm*4 clones persisted throughout the seven year period. This phenomenon may be due to the fact that the *emm*12 and *emm*4 clones produced less M protein and were less virulent than the *emm*12*, *emm*1 and *emm*6 clones.

The PFGE study also indicates that each of the six *emm *clones has one predominant PFGE type, except for the *emm*4 clone, which has two major PFGE types (Figure 2). The less prevalent PFGE genotypes of each *emm *clone emerged and quickly disappeared. Even some major PFGE genotypes, such as SPYS16.0026 of the *emm*12* clone, SPYS16.0020 of the *emm*6 clone and SPYS16.0022 of the *emm*1 clone, remained prevalent for only 2–3 years before declining. However, the SPYS16.0013 genotype of the *emm*12 clone did not follow this trend, as it was prevalent throughout 2000–2006 and was most prevalent in 2006. If a newly emerging strain can only prosper in a specific location for a few years, then the *emm*12:SPYS16.0013 strains isolated during two different time periods should be different. These differences may not be detectable by PFGE analysis. Whether bacterial isolates that prevail for two periods become genetically diversified is an interesting subject and may be studied by other genotyping methods, such as single nucleotide polymorphism, by virulence gene detection and by antimicrobial susceptibility testing.

The SPYS16.0026 isolates, with DNA resistant to *Sma*I digestion, were found in three *emm *types, suggesting that they have multiple evolutionary origins. Of the 127 SPYS16.0026 isolates, 125 belonged to the *emm*12 type. The first isolate resistant to *Sma*I digestion was identified in central Taiwan in 1998 and was an *emm*33 type. The *emm*12:SPYS16.0026 strain was detected for the first time in 1999 [7]. Our previous studies indicated that the *emm*12:SPYS16.0026 strain is most likely derived from an *emm*12:SPYS16.0013 strain by an insertion of a large DNA fragment into the genome [7]. The large DNA segment could have carried the gene(s) responsible for DNA methylation and resistance to cleavage by *Sma*I. These strains were analyzed with *SgrA*I. Clustering analysis of the PFGE-*SgrA*I patterns revealed diverse genetic relationships among the *emm*12:SPYS16.0026 strains (Figure 3). The high genetic divergence suggests that the *emm*12:SPYS16.0026 strains have derived from multiple origins. Recently, Euler et al. [12] have shown that resistance to *Sma*I cleavage is due to the presence of a DNA methyltransferase gene, which is carried on a mobile chimeric element that has transposon- and bacteriophage-like characteristics. This mobile element may explain the high genetic diversity among the *Sma*I-resistant strains that emerged in such short period of time.

The fluctuation of scarlet fever cases between 2000 and 2006 may be partially explained by the shuffling of several prevalent *emm *clones. However, the dramatic drop in reported cases in 2003 is difficult to explain. In early 2003, Taiwan was badly hit by a severe SARS outbreak. The SARS epidemic in Taiwan had two distinct stages, with the beginning in the late-February (the 9^th ^week) and the second ending in mid-June [13]. The stage I epidemic occurred from late-February to mid-April (the 9^th ^to 16^th ^week) and consisted of only scattered, sporadic cases, with most of the patients having recently traveled to China. In this stage, the disease did not cause much panic and the level of scarlet fever remained high. In stage II (from mid-April to mid-June or the 17^th ^to 24^th ^week), several clusters of infection occurred via intra-hospital or inter-hospital transmission. Enormous panic spread over the whole country after an outbreak of nosocomial infection was confirmed on the 22^nd ^of April. The disease was subsequently transmitted to several hospitals and spread from the North to the South. The number of scarlet fever cases dropped remarkably during this period. Because a large portion of the SARS infections was associated with hospitals, fear of SARS drove people out of hospitals and public places. This fear and the change of people's behavior may have significantly reduced the number of outpatients and the transmission of many infectious diseases, including scarlet fever. In fact, the SARS outbreak had a long-term effect on the occurrences of scarlet fever. After the SARS epidemic, the number of weekly scarlet fever reports was often lower than the overall average until the first half of 2004.

## Conclusion

The occurrences of scarlet fever in central Taiwan between 2000 and 2006 were primarily caused by six *emm *clones: *emm*12 (40.0%), *emm*4 (23.2%), *emm*1 (16.3%), *Sma*I-resistant *emm*12* (10.3%), *emm*6 (3.8%) and *emm*22 (2.9%). Each *emm *clone had predominant PFGE genotype(s), and most minor genotypes within an *emm *clone emerged and quickly disappeared. The large fluctuation in the number of scarlet fever cases during this time period can be attributed to the shuffling of several prevalent *emm *clones and to a SARS outbreak in 2003.

## Methods

### Epidemiological data and bacterial strains

Scarlet fever was a notifiable disease in Taiwan until 2007; hospitals and clinics were obligated to report confirmed or suspected cases to the county public health department via a web-based Notifiable Diseases Reporting System established by the Taiwan CDC in 2000. The hospitals and clinics that reported scarlet fever cases were asked to provide throat swab specimens or *S. pyogenes *isolates to the regional laboratories of the Taiwan CDC for bacterial examination and genotyping. Confirmed cases were those in which *S. pyogenes *was isolated from the specimens. The number of annual confirmed cases detected through the Notifiable Diseases Reporting System was adjusted by multiplying the number of reported cases and the rate of positive specimens. *S. pyogenes *isolates used for characterization in this study were obtained directly from hospitals located in central Taiwan through the Notifiable Diseases Reporting System or were recovered from throat swab specimens collected from hospitals and clinics through the Notifiable Diseases Reporting System and the Sentinel Physician Active Reporting System.

### *emm *typing

The procedure developed by Beall and colleagues [5] was used to prepare the *emm *DNA fragments from *S. pyogenes *isolates for sequencing. The amplified DNA amplicons and primer 1, 5'-TATT(C/G)GCTTAGAAAATTAA-3', were sent to a local biotech company (Mission Biotech Corp. Taipei, Taiwan) for DNA sequencing. The 5' *emm *sequences (at least the first 240 bases) were subjected to a BLAST comparison with those in the *emm *database (http://www.cdc.gov/ncidod/biotech/strep/strepindex.htm; accessed on April 20^th^, 2009) to determine *emm *type.

### PFGE analysis

*S. pyogenes *isolates were subjected to PFGE analysis using a previously described protocol [7]. All of the isolates were analyzed by *Sma*I digestion. Isolates with DNA resistant to *Sma*I digestion were analyzed with *SgrA*I. PFGE patterns were recorded using a Kodak digital camera system (Kodak Electrophoresis Documentation and Analysis System 290; Kodak; Rochester, NY, USA) with 1792 × 1200 pixels. The digital PFGE images were then analyzed using BioNumerics software version 4.5 (Applied Maths, Kortrijik, Belgium) and the DNA pattern for each isolate was compared using the computer software. A unique PFGE pattern (genotype) was defined if it contained one or more DNA bands different from the others. The genetic relatedness among isolates is presented in a dendrogram built by clustering the PFGE patterns. The clustering analysis was performed using the UPGMA algorithm provided in the BioNumerics software and the value of Dice predicted similarity of two patterns at settings of 1% optimization and 0.7% position tolerance.

## Authors' contributions

CS Chiou initiated and managed the project, analyzed data and wrote the manuscript. YW Wang worked on *emm *sequencing, PFGE analysis and data analysis. PL Chen collected and analyzed epidemiological data from the Notifiable Diseases Reporting System. WL Wang worked on PFGE analysis. PF Wu coordinated the laboratory and disease surveillance sectors in Taiwan CDC. HL Wei helped with identification of *emm *types. All authors have read and approved the final manuscript.
